# Earth Voices

**Published:** 1889-08-03

**Authors:** 


### EARTH VOICES.

'Goethe's world seems once to have been desolate and baleful as that of the darkest sceptic; but he has covered it anew with beauty and solemnity derived from deeper sources o'er which doubt can have no sway."—Carlyle.

O, toiling earth, your autumn day is dying,
And o'er my soul the night is darkening fast;
The restless wind down dreary streets is sighing,
And bears earth voices on its wailing blast.

Sad undertones from those whose hopes of gladness
Are quenched amid the darkness of this earth,
Moans of sick life, drear cries of wrong and sadness,
And discords ring out loud, above all mirth.

O, painful earth, white feet must tread your winepress,
Bright eyes, while yet they look at light of morn
The light grows grey, and o'er them gathers blackness,
And then young life stands still, all lost and worn.

What is our life? a strange and painful record,
"A glimpse into the world of Might-have-been";
Nay, something more—firm footsteps tending Godward,
And triumphing o'er contradictions mean.

I see white heights across the darkness gleaming,
O'er which grim doubt can hold no dark'ning sway;
We toil through weakness, ignorance, and seeming
Discords, to the clear heights of perfect day.

Dear earth, your midnight fades away, and glistening,
A light comes o'er the darkness, grand and calm;
I hear, with reverent spirit tuned to listening,
Earth's triumph song, I see the victor's palm.

Durham.

